# PKC and CaMK-II inhibitions coordinately rescue ischemia-induced GABAergic neuron dysfunction

**DOI:** 10.18632/oncotarget.16947

**Published:** 2017-04-07

**Authors:** Li Huang, Chun Wang, Shidi Zhao, Rongjing Ge, Sudong Guan, Jin-Hui Wang

**Affiliations:** ^1^ Department of Pathophysiology, Bengbu Medical College, Bengbu 233000, China; ^2^ Department of Endocrinology, The Second Affiliated Hospital of Bengbu Medical College, Bengbu 233040, China; ^3^ Institute of Biophysics, Chinese Academy of Sciences, Beijing 100101, China

**Keywords:** ischemia, GABA, neuron, synapse, PKC and CaMK-II

## Abstract

Cerebral ischemia leads to neuronal death for stroke, in which the imbalance between glutamatergic neurons and GABAergic neurons toward neural excitotoxicity is presumably involved. GABAergic neurons are vulnerable to pathological factors and impaired in an early stage of ischemia. The rescue of GABAergic neurons is expected to be the strategy to reserve ischemic neuronal impairment. As protein kinase C (PKC) and calmodulin-dependent protein kinase II (CaMK-II) are activated during ischemia, we have investigated whether the inhibitions of these kinases rescue the ischemic impairment of cortical GABAergic neurons. The functions of GABAergic neurons were analyzed by whole-cell recording in the cortical slices during ischemia and in presence of 1-[N,O-bis(5-isoquinolinesulfonyl)-N-methyl-L-tyrosyl]-4-phenylpiperazine (CaMK-II inhibitor) and chelerythrine chloride (PKC inhibitor). Our results indicate that PKC inhibitor or CaMK-II inhibitor partially prevents ischemia-induced functional deficits of cortical GABAergic neurons. Moreover, the combination of PKC and CaMK-II inhibitors synergistically reverses this ischemia-induced deficit of GABAergic neurons. One of potential therapeutic strategies for ischemic stroke may be to rescue the ischemia-induced deficit of cortical GABAergic neurons by inhibiting PKC and CaMK-II.

## INTRODUCTION

Ischemic neuron death is presumably initiated by neural excitotoxicity, in which glutamate elevation [1–7] and GABAergic neuron impairment [8–12] have been found to be involved. The elevation of the synaptic glutamate during ischemia may be due to its increased release and impaired reuptake. For instance, the expression of glutamate-transporters changes in ischemia [13–17] and their functions are deficit under pathological conditions [9, 18], such that the impaired glutamate reuptake occurs in ischemia. Moreover, GABAergic neurons are vulnerable to various pathogenic factors and functionally impaired in the early stage of ischemia [9, 12, 19–24]. Their ischemic impairment shifts the balance between excitation and inhibition toward neuronal over-excitation [25], leading to the elevated glutamate release and subsequently neuronal excitotoxicity for ischemic neuronal death. The enhancement of GABAergic synapse transmission reduces loss of hippocampal CA1 pyramidal neurons [26]. In this regard, the elucidation of mechanisms underlying the functional impairment of cerebral GABAergic neurons as well as their protection will provide the clues for developing therapeutic strategies of ischemic neuron death.

Cerebral ischemia triggers a complex series of biochemical and molecular mechanisms that impairs neuronal functions mediated by excitotoxic glutamatergic signaling, ionic balance and free-radical reaction. These intricate processes activate the signaling mechanisms, such as calcium/calmodulin-dependent kinases (CaMK), protein kinase C (PKC) and mitogen-activated protein kinases, which in turn lead to the neuronal stroke [27–31]. In addition to the excitatory neurons, protein kinases, such as PKC and CaMK-II, are located in GABAergic neurons and regulate their functions [32–37]. Based on these studies, we hypothesized that the activated PKC and CaMK-II may be involved in ischemic impairment of GABAergic neurons, and the inhibition of these protein kinases might be able to secure the ischemia-induced deficits of GABAergic neurons.

With questions and hypothesis above, we aim to investigate whether the inhibitions of CaMK-II and PKC are able to prevent the ischemia-induced deficits of GABAergic neurons. In terms of strategies for this study, the functions of cortical GABAergic neurons were measured by whole-cell recording in cortical slices in terms of their abilities of sequential spikes (input-output) and reception (excitatory postsynaptic currents, EPSC). A mimic of cortical ischemia was conducted by reducing perfusion rate to brain slices. The inhibitions of PKC and CaMK-II were fulfilled by applying chelerythrine chloride (PKC inhibitor) and 1-[N,O-bis(5-isoquinolinesulfonyl)-N-methyl-L-tyrosyl]-4-phenylpiperazine (CaMK-II inhibitor).

## RESULTS

### Ischemia attenuates excitatory synaptic transmission and excitability of cortical GABAergic neurons

The functions of GABAergic neurons in cortical slices were measured by whole-cell recording under the conditions of control and subsequent ischemia. Spontaneous excitatory postsynaptic currents (sEPSC) were recorded under voltage-clamp to analyze their receptions to excitatory inputs. Sequential spikes were induced by injecting depolarization pulses in various intensities into these neurons under current-clamp to assess their ability to convert excitatory input into spikes.

**Figure 1:**
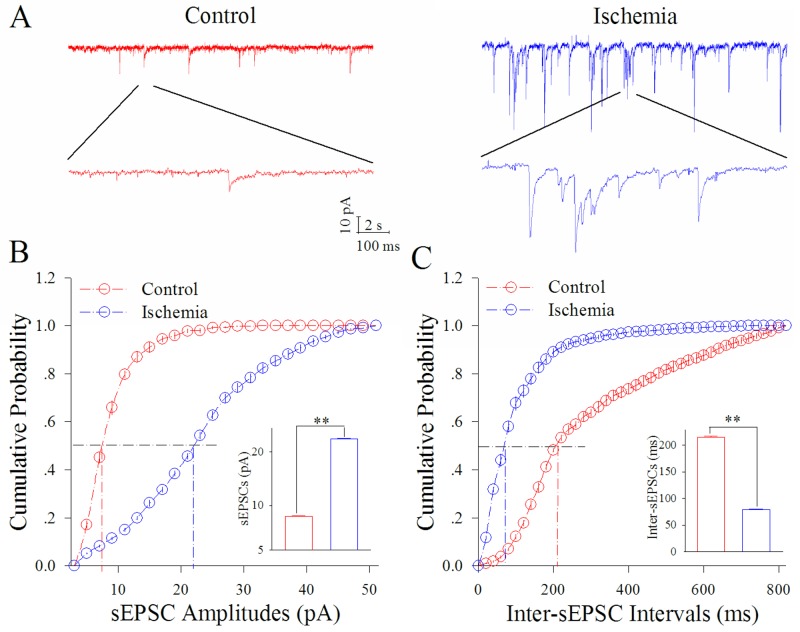
Ischemia upregulates excitatory synaptic transmission at cortical GABAergic neurons sEPSCs were recorded by whole-cell voltage-clamp in cortical GABAergic neurons and analyzed in terms of their amplitudes and frequencies. **(A)** shows sEPSCs recorded in GABAergic neurons during control (red trace) and subsequent ischemia (blue). Calibration bars are 10 pA, 2 seconds (top traces) and 100 ms (bottom trace). **(B)** shows cumulative probability versus sEPSC amplitudes in the control (red symbols) and ischemia (blue). The insert shows sEPSC amplitudes at 50% cumulative probability under the control (red bar) and after ischemia (blue bar; two asterisks, p<0.01; n=13). **(C)** shows cumulative probability versus inter-sEPSC intervals in the control (red symbols) and ischemia (blue). The insert shows that sEPSC intervals at 50% cumulative probability under the control (red bar) and after ischemia (blue bar; two asterisks, p<0.01).

Figure 1 illustrates the ischemia-induced change of sEPSCs on cortical GABAergic neurons. sEPSC amplitudes and frequencies appear to be substantially increased by ischemia (Figure 1A). Figure 1B shows cumulative probability versus sEPSC amplitude in the control (red symbols) and subsequent ischemia (blue, n=13 neurons from 3 mice). Insert figure shows that sEPSC amplitudes at 50% cumulative probability are 8.54±0.21 pA under the control (red bar) and 23.11±0.10 pA after ischemia for 5 min (blue; two asterisks, p<0.01). Figure 1C shows cumulative probability versus inter-sEPSC intervals in the control (red symbols) and subsequent ischemia (blue). Insert figure shows that sEPSC intervals at 50% cumulative probability are 215.27±4.00 ms under the control (red bar) and 78.74±2.13 ms after ischemia for 5 min (blue; two asterisks, p<0.01). This result indicates that ischemia leads to excitatory synaptic transmission at cortical GABAergic neurons to be upregulated.

**Figure 2:**
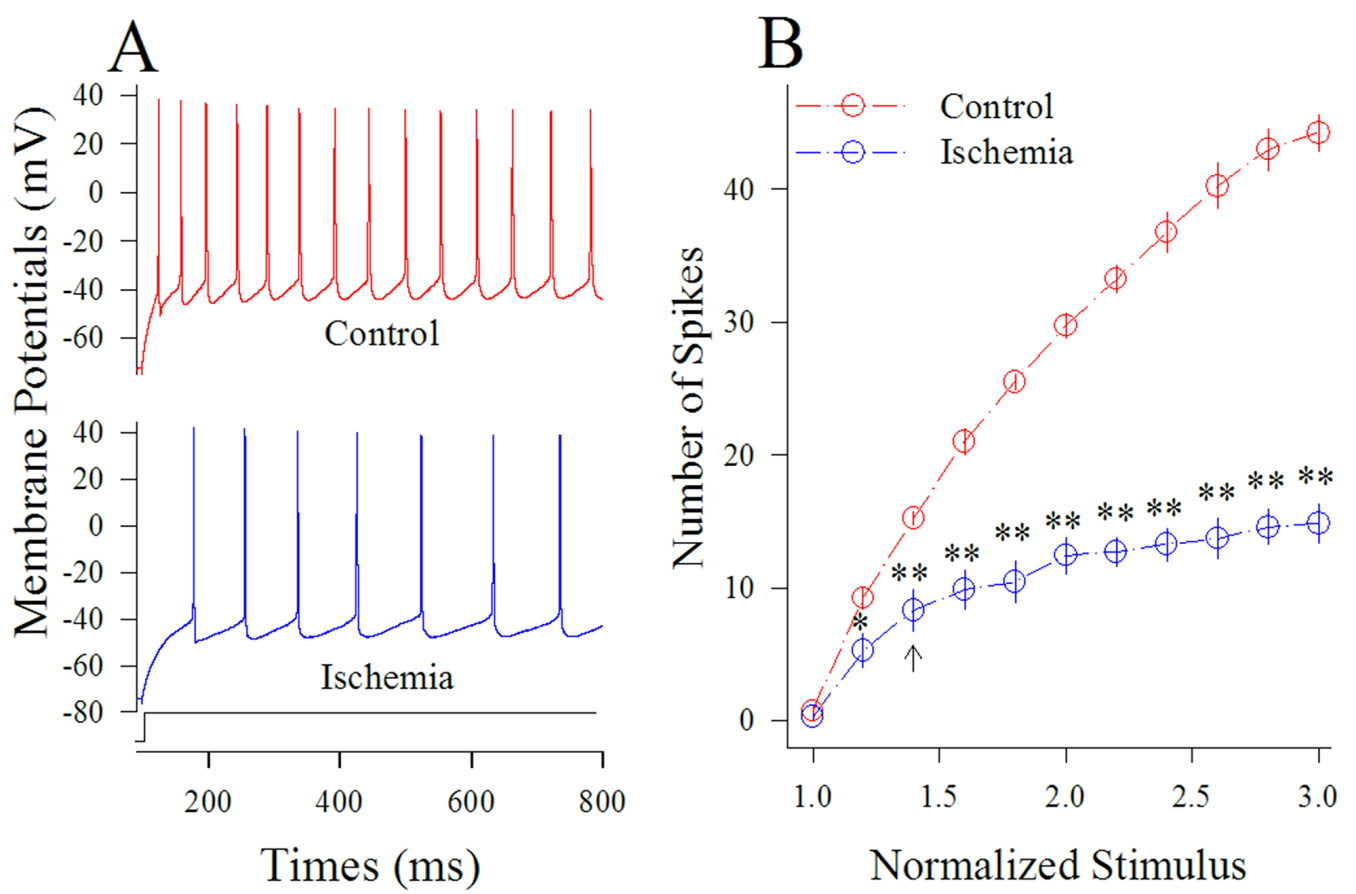
Ischemia impairs spiking ability at cortical GABAergic neurons Sequential spikes were recorded by whole-cell current-clamp in cortical GABAergic neurons and analyzed in terms of spikes per second. **(A)** shows sequential spikes of GABAergic neurons in response to depolarization pulse with the same intensity under the conditions of control (red trace on top panel) and control (blue trace on bottom) at a GABAergic neuron. **(B)** shows that spike frequencies versus normalized stimuli at GABAergic neurons (n=13) under the control (red symbols) and subsequent ischemia (blue; two asterisks, p<0.01).

Figure 2 shows sequential spike capacity at GABAergic neurons under the conditions of control and ischemia. Sequential spikes in Figure 2A were recorded under the conditions of control (top panel) and ischemia (bottom) at a GABAergic neuron in response to the same stimulus intensity of depolarization pulse. Figure 2B shows that spike frequencies versus normalized stimuli at GABAergic neurons (n=13 cells from 3 mice) under the control (red symbols) and subsequent ischemia (blue; two asterisks, p<0.01). This result indicates that spiking ability of cortical GABAergic neurons is substantially impaired after ischemia.

Therefore, ischemia induces the upregulation of excitatory synapses on cortical GABAergic neurons and the downregulation of their spiking ability, indicating ischemic excitotoxicity. If protein kinases, such as CaMK-II and PKC, are involved in this ischemic neuronal impairment, their inhibitors should block the upregulated excitatory synaptic transmission and the downregulated spiking ability, which we have studied.

### The inhibition of CaMK-II partially blocks ischemic over-excitation in cortical GABAergic neurons

The selective inhibitor of CaMK-II, 1-[N,O-bis(5-isoquinolinesulfonyl)-N-methyl-L-tyrosyl]-4- phenylpiperazine (KN-62) [38–40], was used to examine the role of CaMK-II in the ischemic impairment of cortical GABAergic neurons. The experiments were conducted under the conditions of KN-62 presence and subsequent KN-62 plus ischemia.

**Figure 3:**
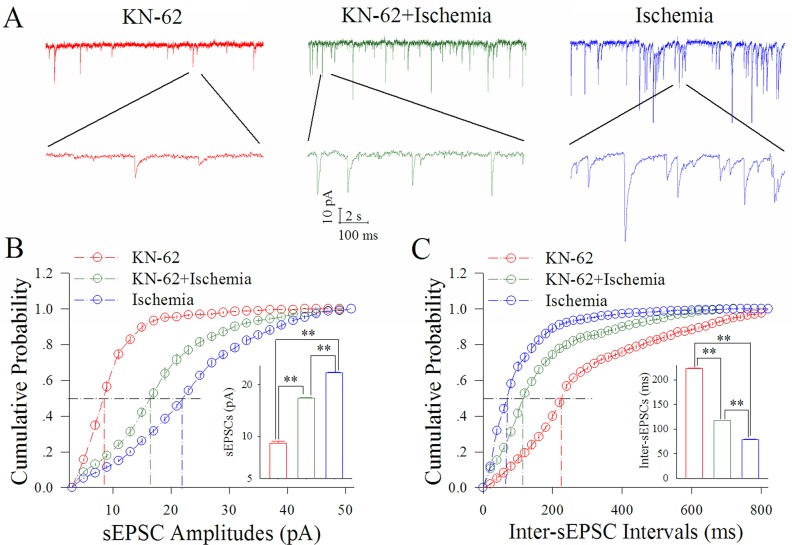
KN-62 partially blocks the ischemic upregulation of excitatory synaptic transmission at cortical GABAergic neurons KN-62 was added into the ACSF with a final concentration at 0.9 μM. **(A)** illustrates sEPSC recorded on GABAergic neurons under the conditions of KN-62 application (red traces), KN-62 plus ischemia (green) and ischemia (blue). **(B)** illustrates cumulative probability versus sEPSC amplitudes under the conditions of KN-62 application (red symbols), KN-62 plus ischemia (green) as well as ischemia (blue). Insert figure shows sEPSC amplitudes at 50% cumulative probability in KN-62 application (red bar), KN-62 plus ischemia (green) and during ischemia only (blue; two asterisks, p<0.01; n=13). **(C)** illustrates cumulative probability versus inter-sEPSC intervals under the conditions of KN-62 application (red symbols), KN-62 plus ischemia (green) and ischemia (blue). Insert figure demonstrates sEPSC intervals at 50% cumulative probability in KN-62 application (red bar), KN-62 plus ischemia (green) and during ischemia only (blue; two asterisks, p<0.01; n=13).

Figure 3 shows the influence of KN-62 on excitatory synaptic transmission at cortical GABAergic neurons in ischemia. KN-62 at 0.9 μM appears to reduce ischemic over-excitation at GABAergic neurons (Figure 3A). Figure 3B illustrates cumulative probability versus sEPSC amplitudes under the conditions of KN-62 application (red symbols) and KN-62 plus ischemia (green; n=13 neurons from 5 mice) as well as ischemia (blue). Insert figure show that sEPSC amplitudes at 50% cumulative probability are 8.97±0.32 pA in KN-62 application (red bar), 17.02±0.12 pA in KN-62 plus ischemia (green) and 23.11±0.10 pA during ischemia only (blue; two asterisks, p<0.01). Figure 3C shows cumulative probability versus inter-sEPSC intervals under the conditions of KN-62 application (red symbols), KN-62 plus ischemia (green) and ischemia (blue). Insert figure shows that sEPSC intervals at 50% cumulative probability are 222.52±2.35 ms in KN-62 application (red bar), 117.21±1.31 ms in KN-62 plus ischemia (green) and 78.74±2.13 ms during ischemia only (blue; two asterisks, p<0.01). Therefore, the inhibition of CaMK-II partially blocks the ischemic upregulation of excitatory synaptic transmission at cortical GABAergic neurons.

**Figure 4:**
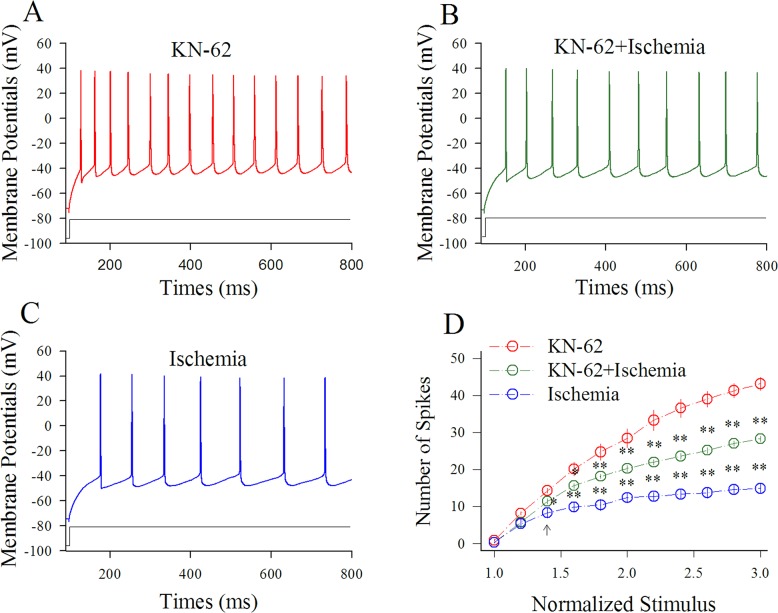
KN-62 prevents the ischemic impairment of spiking capability at cortical GABAergic neurons KN-62 was added into the ACSF with a final concentration at 0.9 μM. **(A)** shows sequential spikes in presence of KN-62 application. **(B)** shows sequential spikes under the condition of KN-62 plus ischemia. **(C)** shows sequential spikes during ischemia. Spikes at the GABAergic neuron in A~C respond to the same stimulus intensity of depolarization pulse. **(D)** shows that spike frequencies versus normalized stimuli at GABAergic neurons (n=13) under the conditions of KN-62 application (red symbols), KN-62 plus ischemia (green) and ischemia (blue; two asterisks, p<0.01).

Figure 4 shows the influence of KN-62 on spiking ability at cortical GABAergic neurons in ischemia. Sequential spikes in Figure 4A-4C were recorded under the conditions of KN-62 application (A), KN-62 plus ischemia (B) and ischemia only (C) at a GABAergic neuron in response to the same stimulus intensity of depolarization pulse. Figure 4D shows that spike frequencies versus normalized stimuli at GABAergic neurons under the conditions of KN-62 application (red symbols) and KN-62 plus ischemia (green; n=13 neurons from 3 mice) as well as ischemia (blue; two asterisks, p<0.01). Thus, the inhibition of CaMK-II partially blocks the ischemic impairment of spiking ability at cortical GABAergic neurons.

These results indicate that CaMK-II plays a role in ischemic over-excitation at cortical GABAergic neurons. We subsequently examine the effect of PKC on the ischemic impairment of cortical GABAergic neurons.

### The inhibition of PKC partially blocks ischemic over-excitation in cortical GABAergic neurons

The selective and potent inhibitor of PKC, chelerythrine chloride (CHE) [41, 42], was used to examine the role of PKC in the ischemic impairment of cortical GABAergic neurons. The experiments were conducted under the conditions of CHE presence and subsequent CHE plus ischemia.

**Figure 5:**
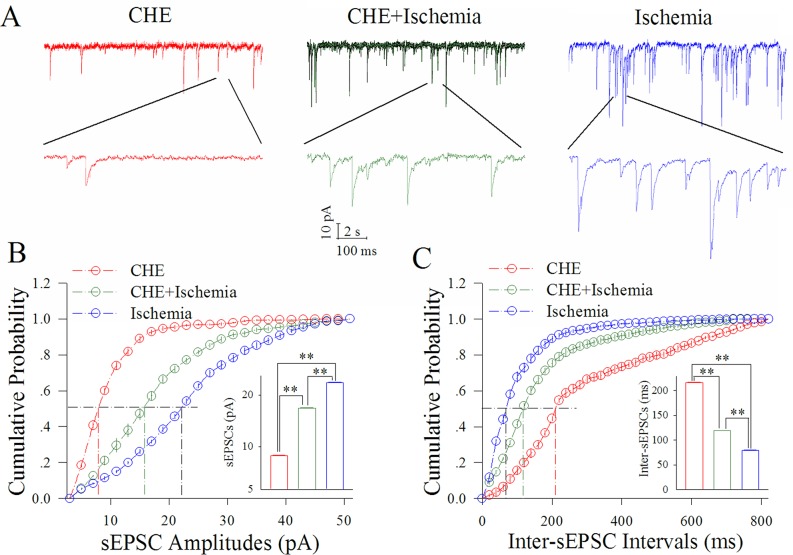
CHE partially blocks the ischemic upregulation of excitatory synaptic transmission at cortical GABAergic neurons CHE was added into the ACSF with a final concentration at 0.6 μM. **(A)** illustrates sEPSC recorded on GABAergic neurons under the conditions of CHE application (red traces), CHE plus ischemia (green) and ischemia (blue). **(B)** illustrates cumulative probability versus sEPSC amplitudes under the conditions of CHE application (red symbols), CHE plus ischemia (green) as well as ischemia (blue). Insert figure shows sEPSC amplitudes at 50% cumulative probability in CHE application (red bar), CHE plus ischemia (green) and during ischemia only (blue; two asterisks, p<0.01; n=13). **(C)** shows cumulative probability versus inter-sEPSC intervals under the conditions of CHE application (red symbols), CHE plus ischemia (green) and ischemia (blue). Insert figure demonstrates sEPSC intervals at 50% cumulative probability in CHE application (red bar), CHE plus ischemia (green) and during ischemia only (blue; two asterisks, p<0.01; n=13).

Figure 5 shows the influence of CHE on excitatory synaptic transmission at cortical GABAergic neurons in ischemia. CHE at 0.6 μM appears to reduce ischemic over-excitation at GABAergic neurons (Figure 5A). Figure 5B illustrates cumulative probability versus sEPSC amplitudes under the conditions of CHE application (red symbols) and CHE plus ischemia (green; n=13 neurons from 4 mice) as well as ischemia (blue). Insert figure shows that sEPSC amplitudes at 50% cumulative probability are 8.69±0.30 pA in CHE application (red bar), 16.99±0.24 pA in CHE plus ischemia (green) and 23.11±0.10 pA during ischemia only (blue; two asterisks, p<0.01). Figure 5C shows cumulative probability versus inter-sEPSC intervals under the conditions of CHE application (red symbols), CHE plus ischemia (green) and ischemia (blue). Insert figure shows that sEPSC intervals at 50% cumulative probability are 216.21±2.37 ms in CHE application (red bar), 118.68±0.95 ms in CHE plus ischemia (green) and 78.74±2.13 ms during ischemia only (blue; two asterisks, p<0.01). Thus, the inhibition of PKC partially blocks the ischemic upregulation of excitatory synaptic transmission at cortical GABAergic neurons.

**Figure 6:**
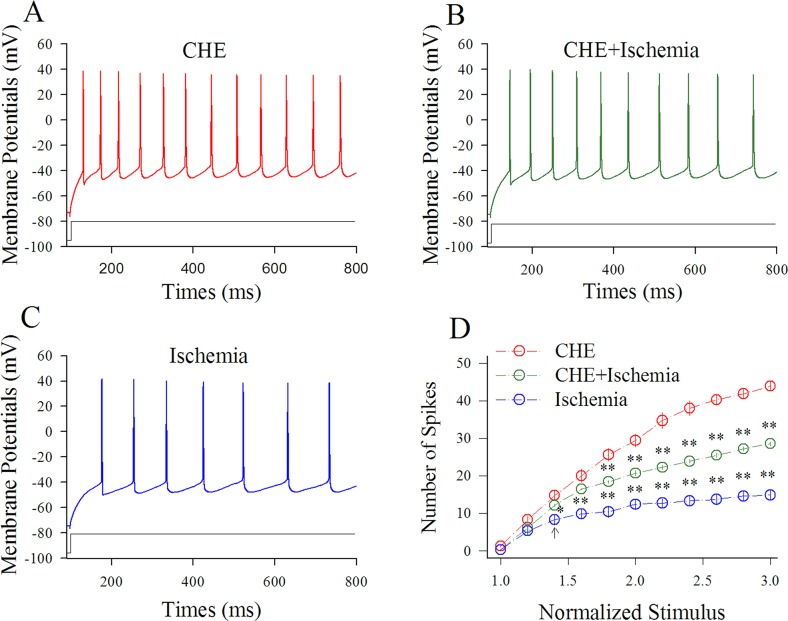
CHE prevents the ischemic impairment of spiking capability at cortical GABAergic neurons CHE was added into the ACSF with a final concentration at 0.6 μM. **(A)** shows sequential spikes in presence of CHE application. **(B)** shows sequential spikes under the condition of CHE plus ischemia. **(C)** shows sequential spikes during ischemia. Spikes at the GABAergic neuron in A~C respond to the same stimulus intensity of depolarization pulse. **(D)** shows that spike frequencies versus normalized stimuli at GABAergic neurons (n=13) under the conditions of CHE application (red symbols), CHE plus ischemia (green) and ischemia (blue; two asterisks, p<0.01).

Figure 6 shows the influence of CHE on spiking ability at cortical GABAergic neurons in ischemia. Sequential spikes in Figure 6A-6C were recorded under the conditions of CHE application (A), CHE plus ischemia (B) and ischemia only (C) at a GABAergic neuron in response to the same stimulus intensity of depolarization pulse. Figure 6D shows that spike frequencies versus normalized stimuli at GABAergic neurons under the conditions of CHE application (red symbols) and CHE plus ischemia (green; n=13 cells from 4 mice) as well as ischemia (blue; two asterisks, p<0.01). Therefore, the inhibition of PKC partially blocks the ischemic impairment of spiking ability at cortical GABAergic neurons.

The results indicate that PKC plays a role in ischemic over-excitation at cortical GABAergic neurons. We subsequently test whether PKC and CaMK-II synergistically block the ischemic impairment of cortical GABAergic neurons.

### The inhibitions of PKC and CaMK-II synergistically block ischemic over-excitation in GABAergic cells

The joint application of CaMK-II and PKC inhibitors was used to examine the role of CaMK-II and PKC in the ischemic impairment of cortical GABAergic neurons. The experiments were conducted under the conditions of KN-62/CHE presence and subsequent KN-62/CHE plus ischemia.

**Figure 7:**
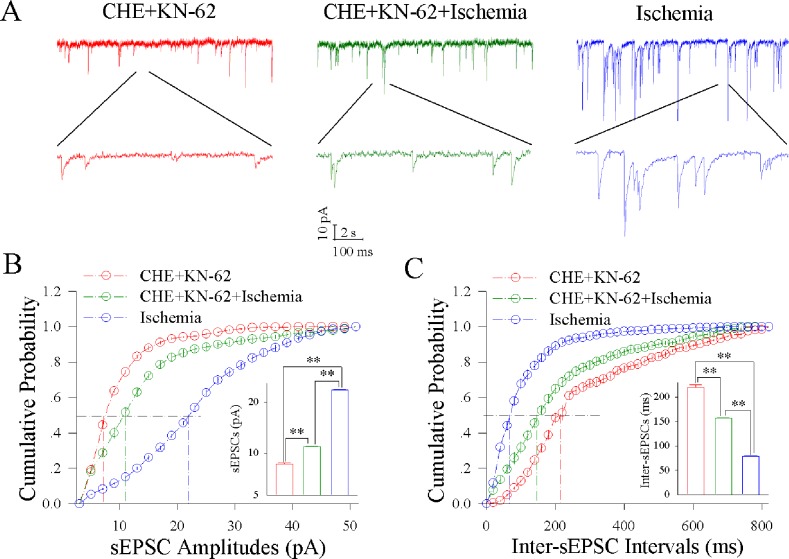
CHE and KN-62 synergistically block ischemic upregulation of excitatory synaptic transmission at cortical GABAergic neurons Final concentrations of CHE and KN-62 in the ACSF are 0.6 μM and 0.9 μM, respectively. **(A)** shows sEPSC recorded on GABAergic neurons under the conditions of CHE/KN-62 application (red traces), CHE/KN-62 plus ischemia (green) and ischemia (blue). **(B)** illustrates cumulative probability versus sEPSC amplitudes under the conditions of CHE/KN-62 application (red symbols), CHE/KN-62 plus ischemia (green) and ischemia (blue). Insert figure illustrates sEPSC amplitudes at 50% cumulative probability in CHE/KN-62 application (red bar), CHE/KN-62 plus ischemia (green) and during ischemia (blue; two asterisks, p<0.01; n=13). **(C)** shows cumulative probability versus inter-sEPSC intervals under the conditions of CHE/KN-62 application (red symbols), CHE/KN-62 plus ischemia (green) and ischemia (blue). Insert figure shows sEPSC intervals at 50% cumulative probability in CHE/KN-62 application (red bar), CHE/KN-62 plus ischemia (green) and during ischemia (blue; two asterisks, p<0.01; n=13).

Figure 7 illustrates the influence of KN-62/CHE on excitatory synaptic transmission at the cortical GABAergic neurons during ischemia. The joint application of KN-62 (0.9 μM) and CHE (0.6 μM) appears to synergistically reduce ischemic over-excitation at GABAergic neurons (Figure 7A). Figure 7B illustrates cumulative probability versus sEPSC amplitudes under the conditions of KN-62/CHE applications (red symbols) and KN-62/CHE plus ischemia (green; n=13 neurons from 5 mice) as well as ischemia (blue). Insert figure shows that sEPSC amplitudes at 50% cumulative probability are 8.46±0.26 pA in KN-62/CHE applications (red bar), 10.99±0.27 pA in KN-62/CHE plus ischemia (green) and 23.11±0.10 pA during ischemia (blue; two asterisks, p<0.01). Figure 7C shows cumulative probability versus inter-sEPSC interval under the conditions of KN-62/CHE applications (red symbols), KN/62/CHE plus ischemia (green) and ischemia (blue). Insert figure shows that sEPSC intervals at 50% cumulative probability are 220.10±11.84 ms in KN-62/CHE applications (red bar), 156.43±3.14 ms in KN-62/CHE plus ischemia (green) and 78.74±2.13 ms during ischemia only (blue; two asterisks, p<0.01). Therefore, the inhibitions of CaMK-II and PKC synergistically blocks the ischemic upregulation of excitatory synaptic transmission at cortical GABAergic neurons.

**Figure 8:**
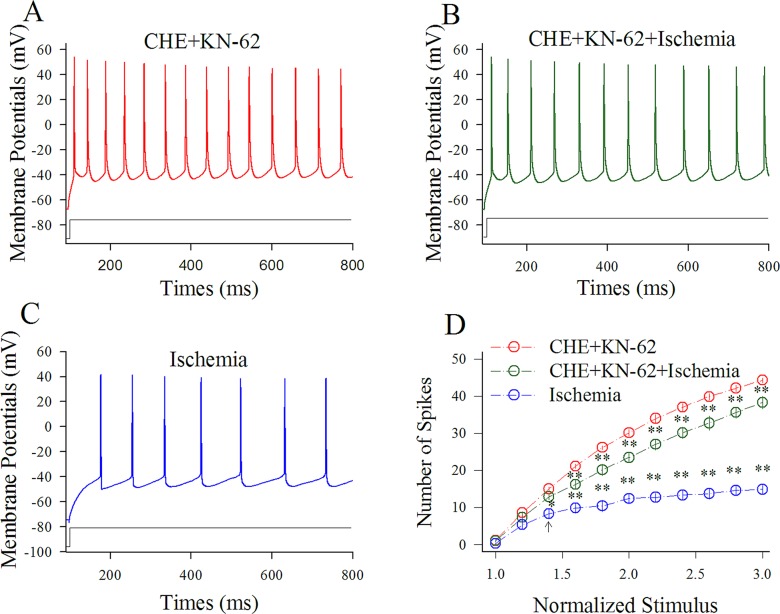
CHE and KN-62 synergistically prevent the ischemic impairment of spiking capability at cortical GABAergic neurons Final concentrations of CHE and KN-62 in the ACSF are 0.6 μM and 0.9 μM, respectively. **(A)** illustrates sequential spikes in presence of CHE/KN-62 application. **(B)** illustrates sequential spikes under the condition of CHE/KN-62 plus ischemia. **(C)** illustrates sequential spikes during ischemia. Spikes at the GABAergic neuron in A~C respond to the same stimulus intensity of depolarization pulse. **(D)** shows that spike frequencies versus normalized stimuli at GABAergic neurons (n=13) under the conditions of CHE/KN-62 application (red symbols), CHE/KN-62 plus ischemia (green) and ischemia only (blue; two asterisks, p<0.01).

Figure 8 shows the influence of KN-62/CHE on spiking ability at cortical GABAergic neurons in ischemia. Sequential spikes in Figure 8A-8C were recorded under the conditions of KN-62/CHE application (A), KN-62/CHE plus ischemia (B) and ischemia only (C) at a GABAergic neuron in response to the same stimulus intensity of depolarization pulse. Figure 8D shows that spike frequency versus normalized stimuli at GABAergic neurons under the conditions of KN-62/CHE application (red symbols) and KN-62/CHE plus ischemia (green; n=13 cells from 5 mice) as well as ischemia (blue; two asterisks, p<0.01). Therefore, the inhibitions of CaMK-II and PKC synergistically blocks the ischemic impairment of spiking capability at the cortical GABAergic neurons.

## DISCUSSION

Our study demonstrates that the ischemia induces the overexcited impairment of cortical GABAergic neurons (Figures 1–2), consistent to previous studies [8–10, 12], which may lead to the excitotoxicity of GABAergic neurons in the early stage of ischemia and subsequently ischemic death of all neurons for cerebral stroke. In terms of molecular mechanisms underlying their impairment, our study indicates that the inhibition of PKC or CaMK-II blocks the ischemic impairment of cortical GABAergic neurons partially (Figures 3–6). The joint application of PKC and CaMK-II inhibitors blocks these pathological changes further. Thus, PKC and CaMK-II prevent the functional impairment of cortical GABAergic neurons synergistically, and the joint application of PKC and CaMK-II inhibitors may be a potential therapeutic strategy to protect cortical neurons from ischemic death.

In our study, ischemia upregulates sEPSC amplitudes and frequencies as well as downregulates spike capability. As we known, sEPSC amplitudes are influenced by the density and conductance of postsynaptic ionotropic glutamate receptors, and sEPSC frequencies are affected by the number and release probability of presynaptic terminals [43, 44]. Moreover, spike ability is influenced by voltage-gated sodium channels [45–48]. In this regard, a sequence of chain reactions for the ischemic dysfunction of cortical GABAergic neurons may be that ischemia leads to intracellular Ca^2+^ increase [8] as well as PKC and CaMK-II activations [49, 50], which further upregulate the glutamate receptor-channels and glutamate release as well as downregulate voltage-gated sodium channels [36, 51–53], such that these GABAergic neurons are dysfunction. In addition to these signaling pathways for the ischemic deficit of cortical GABAergic neurons, other mechanisms remain to be studied.

Our previous study indicates that an impairment of cortical GABAergic neurons induced by ischemia and acidosis is also due to the ischemic dysfunction of glutamate transporters on astrocytes [9]. As the neuron-glia interaction is critical to fulfill brain functions [54–61], the astrocytes may play an important role in the ischemia-induced impairment of cortical GABAergic neurons. Together these data, we suggest that both intracellular signaling pathways and extracellular glia cell supports are critical for protecting GABAergic neurons from ischemic neuron death. In other words, multi-target therapy can also be used in the protection of ischemia neuron injury [62].

In terms of treatment of ischemic stroke, therapeutic strategies include anticoagulation, thrombolysis and neuroprotection [63–73]. Based on the preclinical studies about ischemia-related molecules and biochemical reactions in brain cells, many approaches to interrupt injurious cellular and molecular processes were applied to the clinical trials [74–76]. These efforts have not shown to fully improve stroke patients [1, 4, 74, 75]. In addition to anticoagulation and thrombolysis, the protection of ischemic neuron death by improving multiple targets is needed, since numerous signaling pathways are activated during ischemia. However, the functions of the GABAergic neurons and astrocytes are impaired immediately after ischemia [9, 62]. The protection of neuronal functions in the early stage of ischemia should be focused on the astrocytes and GABAergic neurons in the cerebral cortex. Based on our studies, the joint applications of PKC and CaMK-II inhibitors as well as glutamate transporter activator in a low dosage may be a potential way to pursue.

## MATERIALS AND METHODS

### Cortical slices and GABAergic neurons

The experimental procedures were approved by Institutional Animal Care Use Committee in Bengbu Anhui China. The cortical slices (400 μm) were prepared from C57 GAD-GFP mice (Jackson Lab, USA) whose cortical GABAergic neurons are genetically labeled by green fluorescent protein [77]. The mice in postnatal days 19~21 were anesthetized by inhaling isoflurane and decapitated by a guillotine. Cortical slices were cut with a Vibratome in the oxygenated (95% O_2_ and 5% CO_2_) artificial cerebrospinal fluid (ACSF) in the concentrations (mM) of 124 NaCl, 3 KCl, 1.2 NaH_2_PO_4_, 26 NaHCO_3_, 0.5 CaCl_2_, 4 MgSO_4_, 10 dextrose and 5 HEPES (pH 7.35) at 4°C. The brain slices were held in the oxygenized ACSF (124 NaCl, 3 KCl, 1.2 NaH_2_PO_4_, 26 NaHCO_3_, 2.4 CaCl_2_, 1.3 MgSO_4_, 10 dextrose and 5 HEPES, pH 7.35) at 25°C for 1 hour. A slice was then transferred to the submersion chamber (Warner RC-26G) that was perfused with the oxygenated ACSF at 31°C for whole-cell recording [36, 78–82]. Chemical reagents were purchased from Sigma.

GFP-labeled GABAergic neurons were recorded by whole-cell clamp in the sensory cortices under DIC-fluorescent microscope (Nikon FN-E600, Tokyo, Japan). A wavelength at 488 nm was used to excite the fluorescence of GFP-labeled neurons. GABAergic neurons expressed fast spikes with less adaptation in their amplitude and frequency, the typical properties for the interneurons [83–86].

### Whole-cell recording and neuronal functions

The neurons were recorded by a MultiClamp-700B amplifier under voltage-clamp for their synaptic activity and the current-clamp for their intrinsic properties. Electrical signals were inputted to pClamp-10 (Axon Instrument Inc.) for data acquisition and analysis. An output bandwidth of this amplifier was set at 3 kHz. The pipette solution of recording spikes and excitatory synaptic events included (mM) 150 K-gluconate, 5 NaCl, 5 HEPES, 0.4 EGTA, 4 Mg-ATP, 0.5 Tris-GTP, and 5 phosphocreatine (pH 7.35; [79, 87–89]. These pipette solutions were freshly made and filtered (0.1 μm). The osmolarity was 295~305 mOsmol and pipette resistance was 5~6 MΩ [20, 77].

The functions of GABAergic neurons were assessed by recording their active intrinsic properties (spiking ability) and excitatory synaptic events (reception to excitatory input) [12, 22, 81]. Their excitatory inputs were analyzed by recording spontaneous excitatory postsynaptic currents (sEPSC) on GABAergic neurons in the presence of 10 μM bicuculline in the ACSF to block GABA_A_R [12, 81]. 10 μM CNQX and 40 μM D-AP5 were added in the ACSF at the end of experiments to examine whether synaptic responses were mediated by glutamate receptor, which blocked sEPSCs in our studies.

The recording of spontaneous postsynaptic currents, instead of evoked synaptic currents, is based on the following reasons. sEPSC and sIPSC amplitudes represent the responsiveness and the densities of postsynaptic receptors. The frequencies imply the probability of transmitter release from an axon terminal and the number of presynaptic axons innervated on the recorded neurons [43, 44]. These parameters can be used to analyze presynaptic and postsynaptic mechanisms. Evoked postsynaptic currents cannot separate these mechanisms. We did not apply TTX into the ACSF to record miniature postsynaptic currents since we had to record neuronal excitability. The synaptic events in our recording are presumably miniature postsynaptic currents. This point is supported by a single peak of the postsynaptic currents in our study.

Action potentials at GABAergic neurons were induced by injecting depolarization pulse under the current-clamp. Their excitability was assessed by input-outputs (spikes versus normalized stimuli) when various stimuli were given [24, 90, 91]. We did not measure a rheobase to show cellular excitability, since this strength-duration relationship was used to assess the ability to fire single spike. We measured the ability of firing sequential spikes [80, 92].

Data were analyzed if the recorded neurons had resting membrane potentials negatively more than -60 mV and action potential amplitudes more than 90 mV. Criteria for the acceptance of each experiment also included less than 5% changes in resting membrane potential, spike magnitude, and input resistance throughout each recording. Series and input resistances in all of the neurons were monitored by injecting hyperpolarization pulses (5 mV/50 ms), as well as calculated by voltage pulses versus instantaneous and steady-state currents. The values in the amplitudes and inter-event intervals of sEPSCs were read at 50% of cumulative probability (P_0.5_) for their statistical comparisons [93]. It is noteworthy that sEPSC frequencies were applied to merit presynaptic transmitter release and sEPSC amplitudes were used to merit postsynaptic receptor functions [24].

### *In vitro* ischemia

To simulate the artery occlusion and intracranial anastomotic circulation during *in vivo* ischemic stroke, we reduced the perfusion rate to cortical slices from 2 ml/min to 0.2 ml/min for 6 min [8, 9, 12]. We measured the functions of GABAergic neurons before and during reducing perfusion rate. Subsequently, the perfusion rate was reinstalled to the normal rate before an obvious decrease of resting membrane potentials. In the experiments to examine the influences of protein kinase C (PKC) and Ca^2+^/CaM-dependent protein kinase II (CaMK-II) on neuronal functions, the procedures were the perfusion of the oxygenized ACSF at 2 ml/min for 5 min, the perfusion of the mixture of the oxygenized ACSF plus the inhibitors of PKC and/or CaMK-II at 2 ml/min, and the perfusion of this mixture solution at 0.2 ml/min.

The effects of PKC on sEPSC and spiking ability in GABAergic neurons and their ischemia-induced deficit were examined by using its selective and potent inhibitor, chelerythrine chloride (CHE IC_50_=0.6 μM; Sigma, USA) [41, 42], which lowered PKC activity [94–97]. CHE was dissolved in Dimethyl Sulphoxide with a concentration at 0.6 μM. The influences of CaMK-II on sEPSC and spiking ability in cortical GABAergic neurons and their ischemia-induced deficit were examined by applying its selective inhibitor, 1-[N,O-bis (5-isoquinolinesulfonyl)-N-methyl-L-tyrosyl]-4-phenylpiperazine (KN-62; IC_50_=0.9 μM; Sigma, USA) [38–40]. KN-62 was dissolved in Dimethyl Sulphoxide with concentration at 0.9 μM. As the concentrations of CHE and KN-62 being used in our study were 0.6 and 0.9 μM, respectively, i.e., IC50, such low concentrations were thought to be specific. Moreover, these concentrations of reagents do not affect basal synaptic transmission and neuronal spiking ability (Supplementary Figure 1).

### Statistical analyses

The data of electrophysiological recordings are presented as mean±SEM. The paired t-test was used in the comparisons of experimental data before and after the ischemia or kinase inhibitor application in each of the mice. One-way ANOVA was used to make statistical comparisons in neuronal activity among control, PKC inhibitor, CaMK-II inhibitor and their mixtures.

## SUPPLEMENTARY MATERIALS FIGURES AND TABLES


