# Supplementary material for: PKC and CaMK-II inhibitions coordinately rescue ischemia-induced GABAergic neuron dysfunction
Source: Oncotarget. 2017 Apr 7;8(24):39309–22. doi: 10.18632/oncotarget.16947 (PMC5503615; doi:10.18632/oncotarget.16947)
Supplement: Supplementary file 1 [file oncotarget-08-39309-s001.pdf]

PKC and CaMK-II inhibitions coordinately rescue ischemia-induced GABAergic neuron dysfunction

SUPPLEMENTARY MATERIALS

SUPPLEMENTARY FIGURE

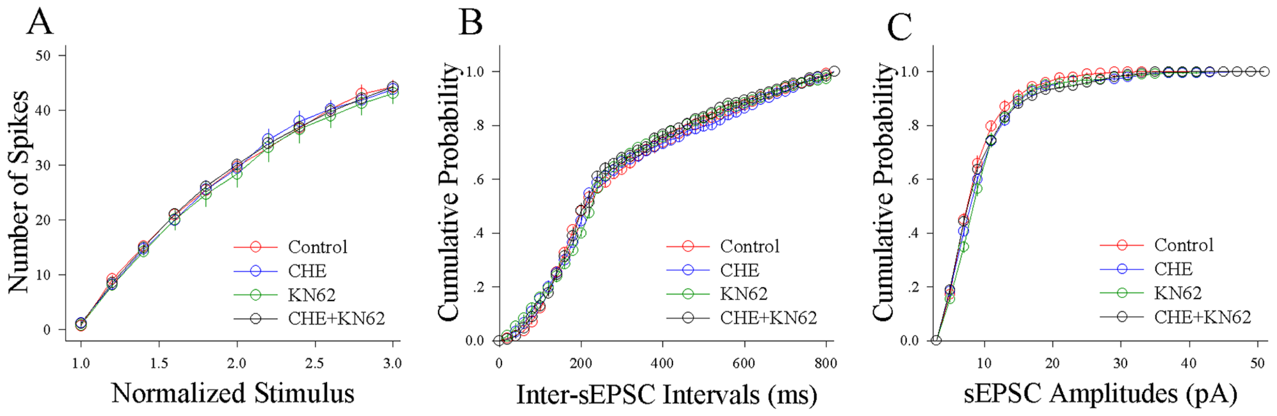

Supplementary Figure 1: The effects of PKC and CaMK-II inhibitors on basal synaptic transmission and neuronal encoding. (A) shows spikes versus normalized stimuli under the conditions of control (red symbols), CHE (blue), KN-62 (green) and CHE plus KN-62 (dark). **(B)** shows cumulative probability versus inter-sEPSC intervals under the conditions of control (red symbols), CHE (blue), KN-62 (green) and CHE plus KN-62 (dark). **(C)** shows cumulative probability versus sEPSC amplitudes under the conditions of control (red symbols), CHE (blue), KN-62 (green) and CHE plus KN-62 (dark).
